# Altered Cervical Vestibular-Evoked Myogenic Potential in Children with Attention Deficit and Hyperactivity Disorder

**DOI:** 10.3389/fneur.2017.00090

**Published:** 2017-03-13

**Authors:** Valeria Isaac, Diego Olmedo, Francisco Aboitiz, Paul H. Delano

**Affiliations:** ^1^Otolaryngology Department, Clinical Hospital of the University of Chile, Santiago, Chile; ^2^Pediatric Diagnostic and Therapy Center, CERIL, Santiago, Chile; ^3^Departamento de Psiquiatría and Centro Interdisciplinario de Neurociencia, Pontificia Universidad Católica de Chile, Santiago, Chile; ^4^Physiology and Biophysics, ICBM, Faculty of Medicine, University of Chile, Santiago, Chile

**Keywords:** VEMP, attention deficit and hyperactivity disorder, subjective visual vertical, otolith function, balance, gait

## Abstract

**Objective:**

Emerging evidence suggests that children with attention deficit and hyperactivity disorder (ADHD) present more difficulties in standing and walking balance than typically developing children. Most of previous studies have assessed these functions using postural and sensory organization tests showing differences in balance performance between control and ADHD children. However, to date, it is unknown whether these balance alterations are accompanied with vestibular dysfunction. The principal aim of this study is to evaluate vestibular otolith function in ADHD and matched control children.

**Methods:**

We assessed vestibular otolith function in children with ADHD and controls using the subjective visual vertical (SVV) bucket test and cervical vestibular-evoked myogenic potentials (cVEMPs). In addition, gait and balance were evaluated using the dynamic gait index (DGI) and computerized posturography.

**Results:**

Non-significant differences between groups were obtained in SVV evaluation. DGI results show lower scores for overall test performance in children with ADHD (*p* < 0.001), while computerized postural recordings showed significant differences for the limit of stability between groups (*p* = 0.02). cVEMPs in response to 500 Hz tone bursts presented at 100 dB were absent or reduced in children with ADHD, as revealed by differences in P1 and N1 peak-to-peak amplitudes between groups (*p* < 0.01).

**Conclusion:**

These findings suggest that vestibular brainstem reflexes are altered in a subset of children with ADHD. We propose to include cVEMP reflexes in the clinical evaluation of ADHD patients.

## Introduction

Attention deficit and hyperactivity disorder (ADHD) is a neuropsychiatric condition characterized by the presence of inattention, hyperactivity, and impulsivity (1) associated to disturbances in the maturation process of executive functions (2). However, this diagnosis is based exclusively on behavioral symptoms that may result from a wide range of underlying causes and susceptibilities (3). Great research efforts have been devoted to unravel the physiopathology of this group of symptoms (4) being one of the most currently accepted theories the deficit in dopamine-signaling mechanisms affecting prefrontal cortex, basal ganglia, and amygdala circuits, which participate in executive functions (5). In addition, these dopamine-signaling deficits are associated to genetic factors encoding for dopamine receptor DRD4 and dopamine transporter DAT1 (6).

Previous evidence indicates that a subset of children with ADHD have abnormal gait and balance (7–9). As the cerebellum is an important structure for the control of posture and gait, several authors have searched for cerebellum abnormalities in ADHD patients. For instance, Castellanos et al. (10) found significantly smaller cerebellar hemispheric volumes in ADHD patients. Similarly, Berquin et al. (11) showed that the posterior–inferior lobules of the cerebellum (VIII–X) are significantly smaller in ADHD children, proposing a cerebello–thalamo–prefrontal circuit dysfunction in ADHD. Moreover, Buderath and colleagues (12) observed a reduction in the cerebellar volume of ADHD children with balance disorders using volumetric magnetic resonance imaging and static and dynamic posturography. Together, these studies link ADHD diagnosis with a possible cerebellar dysfunction that may contribute to balance and gait disorders in a subset of ADHD patients.

However, in addition to the cerebellum, the vestibular system is one of the most important neural networks involved in the control of balance and gait. There are five different vestibular receptors, including two otolith organs (utricle and saccule) and three semicircular canals. Semicircular canals detect angular acceleration and project mainly to ocular-motor nuclei in the brain stem, stabilizing the visual field while compensating for head movements through vestibular–ocular reflexes (13). On the other hand, otolith organs detect linear acceleration and send information to cerebellum and spinal cord (vestibular–spinal tracts) related to postural control and balance functions (14). Otolith function influences the ability to maintain a standing upright position while moving along slanted or uneven surfaces (15) without losing balance. Maintaining balance encompasses the acts of achieving postural alignment relative to the base of support and restoring the body center of mass within the limits of stability (LOS) (16). Particularly, the main function of the utricle and saccule is the maintenance of body orientation in space along with stabilizing posture and equilibrium, accomplished by several vestibular reflexes acting on body muscles (14).

The principal aim of this study was to assess vestibular otolith function using cervical vestibular-evoked myogenic potentials (cVEMPs) and the perception of subjective visual vertical (SVV) in ADHD and matched control children. In addition, postural and gait performances were also measured and correlated with cVEMP responses.

## Materials and Methods

### Subjects

Thirteen children with ADHD (mean age 7.8 ± 1.7 years, range between 5 and 10 years, 9 males) and 13 age-matched healthy controls (mean age 7.3 ± 1.5 years, range between 5 and 10 years, 3 males) were enrolled in the study. ADHD participants were selected from the Pediatric and Diagnosis Center Ceril, while control children were recruited from schools with similar socioeconomic level to that of ADHD children in Santiago. All children were known to be full-term born infants and currently assisting regularly to school. All ADHD participants matched the DSM-5 criteria for the combined type (1) and were particularly evaluated and diagnosed by a pediatric neurologist or by a psychiatrist. None of the ADHD children were under pharmacological treatment at the time of testing. The ADHD children had no other neuropsychiatric comorbidities. Written informed consent was obtained from all participating children and parents in accordance with the Declaration of Helsinki. The ethics committee from the Clinical Hospital of the University of Chile approved the study (approval number: OAIC 785/16).

### Behavioral Assessment

In order to assess ADHD symptom severity and differentiate ADHD-like behaviors from healthy controls, we used the sensory processing measure (SPM) main classroom form, designed for children from ages 5 to 12 (17). The SPM was translated to Spanish according to the suggestions made by Su and Parham (18) for cross-cultural use of questionnaires. The 16 items used from the SPM form employ a rating scale based on how frequently behaviors occur, each item is rated in one of four categories: never, occasionally, frequently, or always. A numeric score (1–4) is assigned to each rating, with higher scores indicating more dysfunctional behavior (17). Each child’s main classroom teacher filled out the SPM form. All items were then added up to give a total numeric score going from 0 to 64 points.

### Subjective Visual Vertical

The SVV perception comprises the integration of different sensory modalities: visual, vestibular, and somesthetics (19) within the central nervous system, associated with increased activity in the parietal and occipital cortical areas (20). Previous studies have indicated that graviceptive otolith signals play an important role in the multisensory integration system for the perception of visual verticality (21). The bucket method is an effective and reliable way to determine SVV (22) and is considered to be an indicator of peripheral and central vestibular disorders (23, 24). In this study, we used a modified version of the bucket method to measure the perception of the SVV. The bucket consisted of a metallic cylinder 40-cm long and 25 cm in diameter placed on a manually rotating base. On the bottom, looking inside the bucket, a black thick straight line was presented, and on the bottom outside, a digital sensor was fixed for tilt degree measurements (0.1° degrees of precision). Subjects had to stand facing the bucket looking toward the inside, with their visual fields covered by the rim of the bucket. To ensure that no visual cues were used, a white linen fabric, loosely placed covering the bucket and child’s head, was added. For measurements the bucket was rotated right or left by the examiner, starting from a 45° angle moving toward the 0° position. Subjects were instructed to verbally signal (saying “stop” or “now”) when they estimated the line being vertical. Degrees off the vertical zero were recorded. This procedure was repeated six times for each subject (alternating between clockwise and counterclockwise rotation).

### Dynamic Gait Index (DGI)

The DGI is a performance-based assessment tool developed by Shumway-Cook and Wollacott (25) to quantify dynamic balance abilities during gait task demands (26). Balance during gait is achieved through a complex integration of multiple bodily systems, which include vestibular, proprioceptive, visual, motor, and premotor systems, that takes place in higher processing areas of the central nervous system (16). Studies show that a functional linkage between otolith signals and activity in lower limb muscles is detectable in normal human gait. The otolith input appears to dominate particularly the neck proprioceptive and gaze motor influences during normal gait (27). The DGI has been found to be a sensitive and efficient tool for adults (28) and a feasible and easy to administrate test for children (26). The DGI consists of eight items, and each of these items is scored based on gait balance performance (0–3 points) with a maximum total score of 24 points. These eight items, carefully described for proper administration include (1) walking at normal speed, (2) walking with changes in speed, (3) walking with horizontal head turns, (4) walking with vertical head turns, (5) walking then pivoting, (6) walking and stepping over obstacles, (7) walking around obstacles, and (8) walking up and down stairs. Each item was verbally explained to the children, and items 2–7 were demonstrated as well (items 1 and 8 are not demonstrated since these are scored based on the child’s typical behavior).

### Static Posturography

Postural control during balance was assessed using the balance rehabilitation unit (BRU^®^). Static posturography uses a stable platform set to record body pressure center and body sway velocity (SW). Children were instructed to stand on the platform wearing a pair of virtual reality goggles, while completing 10 given tasks. For the first task (task 0), an area for LOS was established, where the child, standing with both feet on platform and wearing the goggles, is instructed to shift body center of pressure (COP) as far away as possible, in all directions (as indicated), without losing balance and without moving feet. The next five tasks were performed under the condition of “restricted vision,” with the goggles showing a black screen. Tasks under this condition include (1) standing as still as possible on two feet, (2–3) on one foot and then the other foot, (4–5) on tandem switching the foot in front. The following three tasks, (6) standing as still as possible on both feet, (7–8) then on one foot and then the other, are performed under the condition of “visual distractions” (vertical optokinetic stimulation). In the last task (9), the child is presented with a virtual environment and instructed to explore through trunk and head movements but keeping both feet stable on platform. For each of the nine tasks, COP, in square centimeters, and SW, in centimeters per second, were recorded. As children (especially ADHD children) cannot maintain attention during long-lasting evaluations, we had to abbreviate the posturography protocol, and COP areas and SW velocities were not measured with foam.

### Cervical Vestibular-Evoked Myogenic Potentials

Cervical VEMPs were recorded using surface electrodes placed over the sternocleidomastoid (SCM) muscles, while reference and ground electrodes were placed on the upper sternum and in the midline of the forehead, respectively. Subjects sat comfortably on a chair, keeping the SCM activated and tense through head rotation. We used the Eclipse, EP25-Interacoustics^®^ equipment with research license, Denmark. The electromyographic (EMG) signal was amplified and band-pass filtered (10–1,000 Hz), and the rectified EMG signal was measured to obtain valid trials with muscle activation. The stimulus consisted of 500 Hz tone bursts, presented at 5.1 Hz rate through earphones at 100 dB nHL. To obtain cVEMPs waveforms, 200 trials were averaged, and P1–N1 amplitudes and P1 latencies were measured. To rule out middle ear conductive alterations, an otoscopic examination of the tympanic membrane was performed and middle ear impedance was measured.

### Data Analysis

Descriptive statistics including mean ± SEM or median and interquartile range (IQR) were computed for all variables in ADHD and control groups. Normal distribution of data was evaluated using the Shapiro–Wilk test. Non-parametric Mann–Whitney *U* tests were used to compare SVV, DGI, and posturography tasks. VEMP amplitudes and latencies were compared with *t*-tests analyses. Spearman correlation tests were used to evaluate possible association between the different variables. *p*-Values <0.05 were considered as significant in all analyses.

## Results

**Figure 1:**
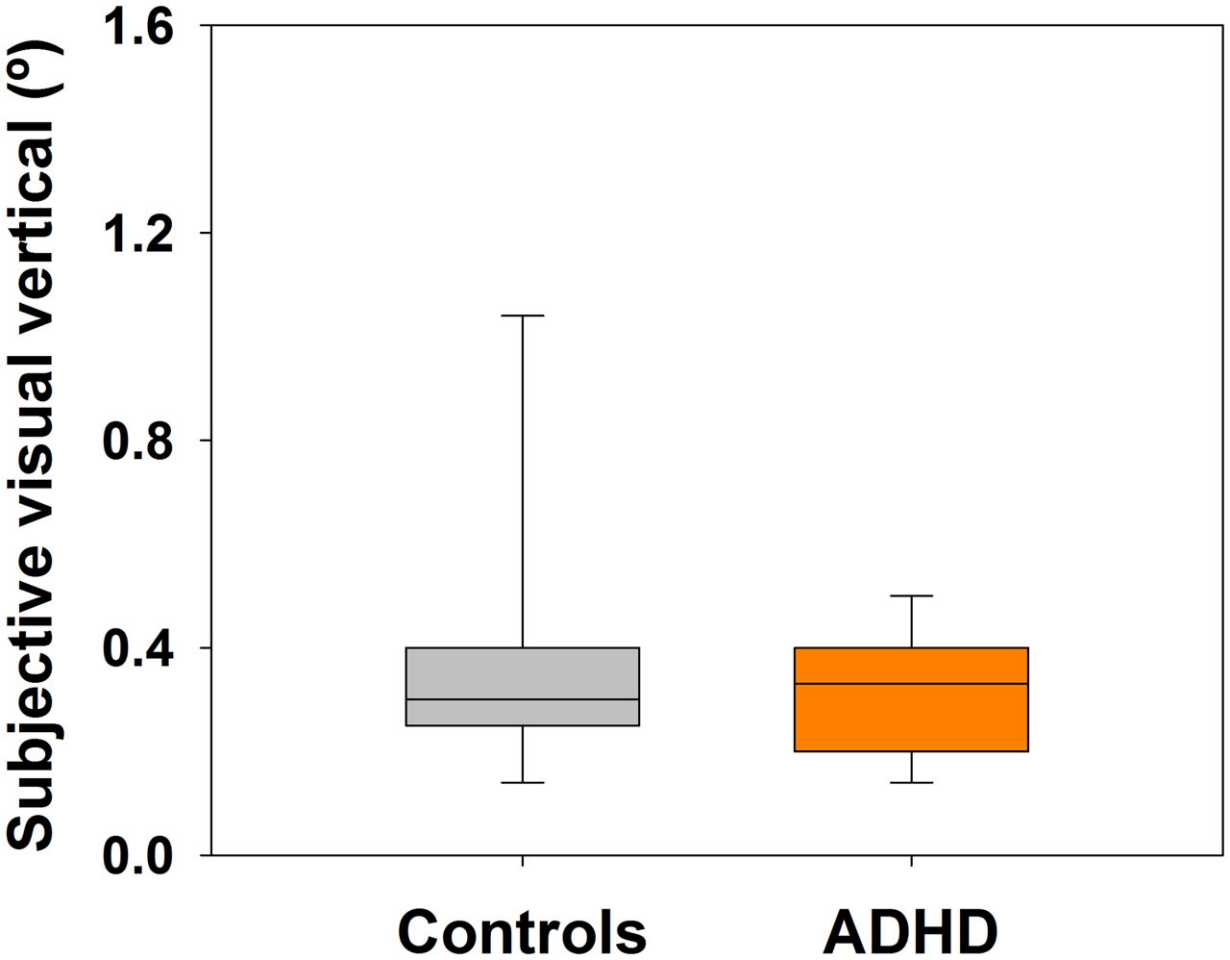
**Similar subjective visual vertical (SVV) perception in controls (*n* = 13) and attention deficit and hyperactivity disorder (ADHD) children (*n* = 13)**. Box-plots showing the median and interquartile range for six attempts in each group. Non-significant differences were observed, revealing similar abilities in both groups for SVV perception.

**Figure 2:**
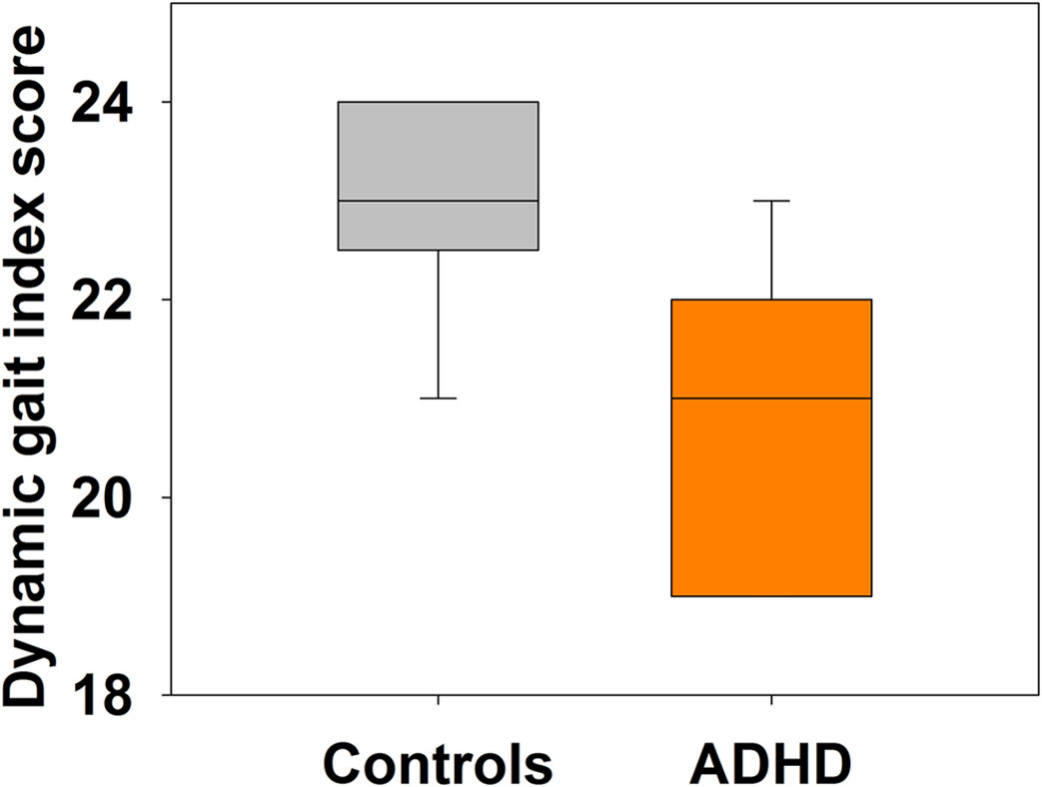
**Attention deficit and hyperactivity disorder (ADHD) children have reduced total dynamic gait index (DGI) score compared to controls**. Mann–Whitney, *p* = 0.0004. Box-plots showing the median and interquartile range for total DGI score in both groups.

Controls had a mean total SPM score of 19.2 ± 0.4 points (mean ± SEM), while children diagnosed with ADHD had a mean total SPM score of 32.5 ± 1.8 points, which was significantly worse for ADHD children than for controls (Mann–Whitney *U* Statistic = 0.0, *p* < 0.001). Regarding SVV perception, there were non-significant differences in the average of SVV between controls (median = 0.3°, IQR: 0.25–0.4°) and ADHD children (median = 0.4°, IQR: 0.2–0.4°) (Mann–Whitney *U* Statistic = 77.5, *p* = 0.731) (Figure 1). All evaluated subjects had an average SVV value in the normal range (below ± 2.5°). On the other hand, DGI scores showed lower scores for overall test performance in children with ADHD (median = 21, IQR: 19–22) compared to controls (median = 23, IQR: 22.5–24) (Mann–Whitney *U* Statistic = 19.0, *p* < 0.001) (Figure 2). Table 1 shows each of the eight tasks used for computing DGI scores. Significant differences between ADHD and controls groups were obtained in tasks 2, 3, 4, and 6, which correspond to changes in gait speed (2), horizontal and vertical head movements during gait (3 and 4), and stepping over obstacles (6). None of the children in the ADHD group scored the maximum of 24 points in the DGI.

**Table 1:** **Dynamic gait index (DGI) in attention deficit and hyperactivity disorder (ADHD) and control children**.

DGI task	Control (mean ± SEM)	ADHD (mean ± SEM)	*p*-Value (Mann–Whitney)
1.Normal gait	3.00 ± 0.00	3.00 ± 0.00	N.S.
2.Changes in gait speed	2.92 ± 0.07	2.39 ± 0.24	*p* = 0.031
3.Horizontal head movements	2.77 ± 0.12	2.00 ± 0.23	*p* = 0.011
4.Vertical head movements	2.69 ± 0.13	2.15 ± 0.19	*p* = 0.039
5.Pivot	2.77 ± 0.12	2.69 ± 0.13	N.S.
6.Stepping over obstacle	3.00 ± 0.00	2.54 ± 0.14	*p* = 0.007
7.Walking around obstacle	2.92 ± 0.08	2.85 ± 0.10	N.S.
8.Stairs	3.00 ± 0.00	3.00 ± 0.00	N.S.
Total DGI score	23.07 ± 0.31	20.77 ± 0.41	*p* < 0.001

**Figure 3:**
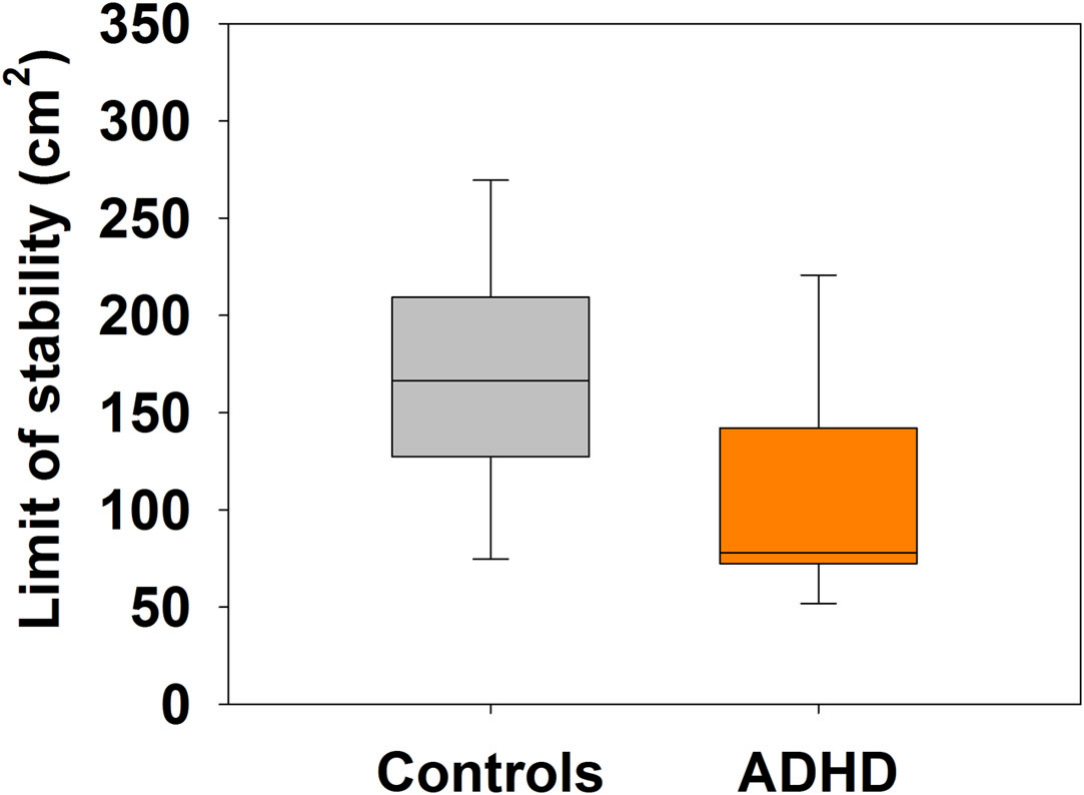
**Attention deficit and hyperactivity disorder (ADHD) children have significant reduced limits of stability (LOS) area compared to control children**. Box-plots showing the median and interquartile range for LOS in both groups.

The area of the LOS measured by computerized posturography showed significant differences between ADHD (median = 77.91 cm^2^, IQR: 72.34–142.06 cm^2^) and control children (median = 166.55 cm^2^, IQR: 127.29–209.45 cm^2^) (Mann–Whitney *U* Statistic = 35.0, *p* = 0.037) (Figure 3). Regarding age and height, there were significant positive correlations with LOS area in the control group (age: Spearman, *R* = 0.547, *p* = 0.049; height: *R* = 0.633, *p* < 0.001), but not in the ADHD group (age: Spearman, *R* = 0.087, *p* = 0.776; height: *R* = 0.187, *p* = 0.557). In addition, we measured the COP and SW in nine situations with different motor and visual conditions (described in Section “Materials and Methods”). Table 2 shows a summary of the performance in the nine tasks, evidencing significant differences in COP for task 1 (keeping balance with both feet on platform with black screen), task 3 [balancing on one foot (non-dominant side) black screen], and task 9 (exploring a panoramic view). Regarding SW, we found significant differences in tasks 1, 3, and 6.

**Table 2:** **Computerized posturography in attention deficit and hyperactivity disorder (ADHD) and control children**.

	Control		ADHD		*p*-Value (Mann–Whitney)
	Center of pressure (COP) (cm^2^)	Sway velocity (SW) (cm/s)	COP (cm^2^)	SW (cm/s)	A: COP B: SW
1.Standing two feet	13.35 ± 5.89	2.01 ± 0.31	17.32 ± 4.92	2.66 ± 0.24	A: *p* = 0.024
					B: *p* = 0.032
2.Standing dominant feet	109.45 ± 28.46	12.06 ± 1.69	189.37 ± 37.15	14.21 ± 1.55	A: N.S.
					B: N.S.
3.Standing non-dominant feet	94.92 ± 23.67	11.42 ± 1.49	230.41 ± 49.46	17.03 ± 1.86	A: *p* = 0.013
					B: *p* = 0.028
4.Tandem dominant	36.50 ± 8.95	5.81 ± 0.76	67.49 ± 26.62	6.76 ± 0.70	A: N.S.
					B: N.S.
5.Tandem non-dominant	44.69 ± 11.53	6.43 ± 0.68	46.84 ± 14.61	7.04 ± 1.10	A: N.S.
					B: N.S.
6.Standing two feet/OPK	7.57 ± 1.62	1.91 ± 0.18	13.00 ± 2.5	2.58 ± 0.17	A: N.S.
					B: *p* = 0.02
7.Standing dominant feet/OPK	116.69 ± 31.03	12.18 ± 1.77	198.90 ± 28.56	14.50 ± 1.31	A: N.S.
					B: N.S.
8.Standing non-dominant feet/OPK	111.61 ± 28.55	11.42 ± 1.05	192.92 ± 47.19	12.90 ± 1.71	A: N.S.
					B: N.S.
9.Virtual panorama	33.79 ± 5.73	4.24 ± 0.61	16.29 ± 1.98	3.40 ± 0.33	A: *p* = 0.011
					B: N.S.

**Figure 4:**
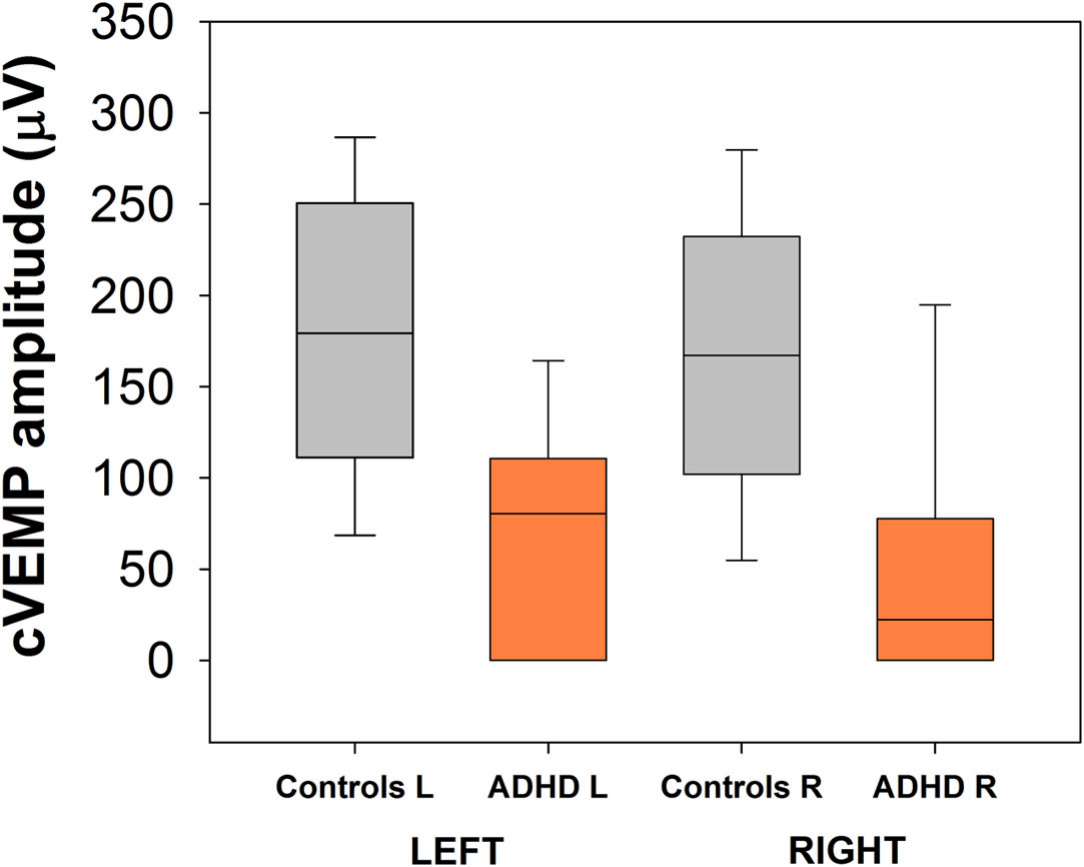
**Bilateral cervical vestibular-evoked myogenic potential (cVEMP) amplitudes are reduced in the group of attention deficit and hyperactivity disorder (ADHD) children**. Significant differences were obtained comparing left and right cVEMPs obtained with 500 Hz tones at 100 dB.

**Figure 5:**
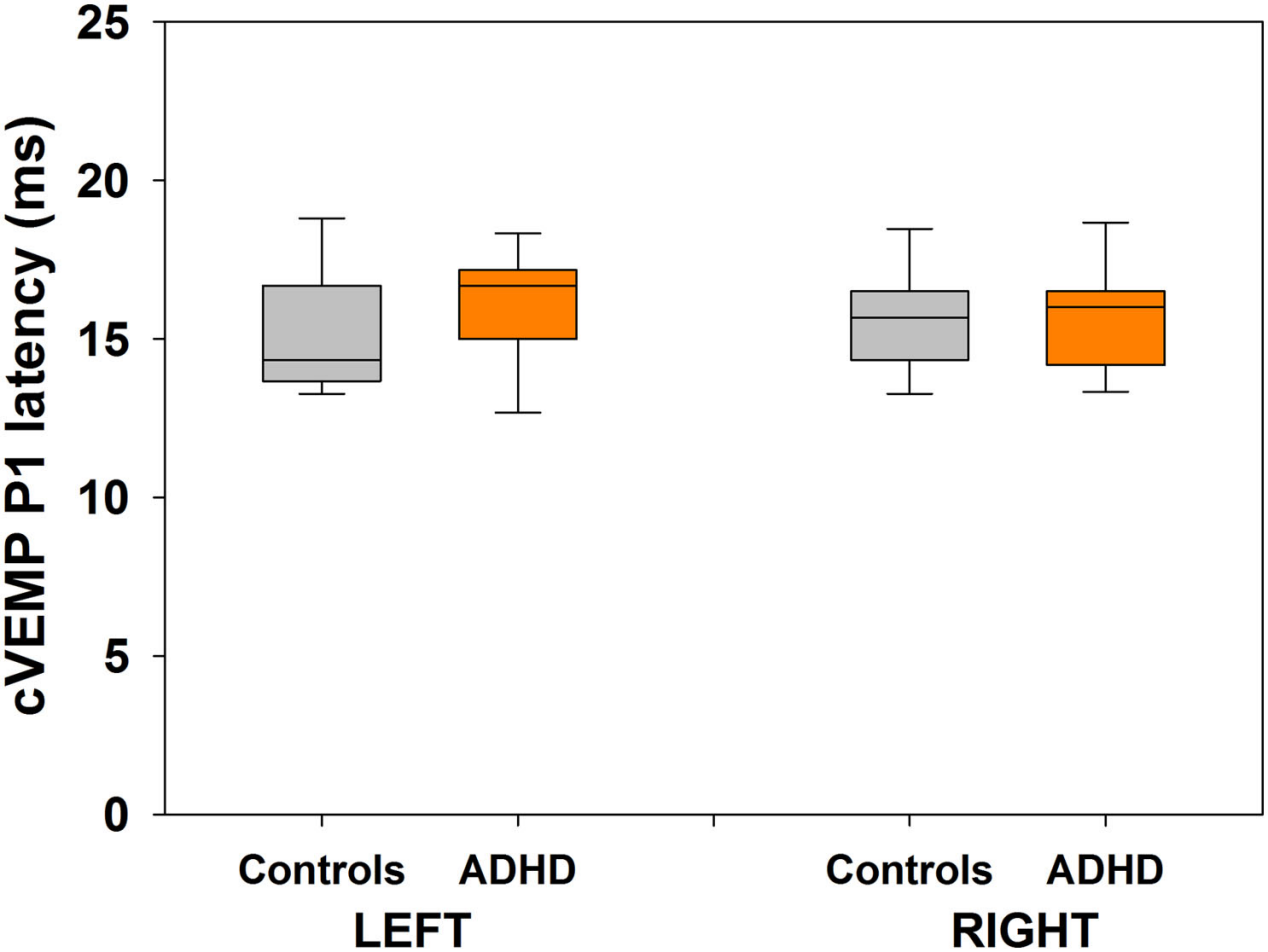
**There are no differences in cervical vestibular-evoked myogenic potential (cVEMP) P1 latencies between the group of attention deficit and hyperactivity disorder (ADHD) and control children**. Box-plots showing the median and interquartile range for P1 latencies in both groups. Left and right cVEMPs were obtained with 500 Hz tones at 100 dB.

**Figure 6:**
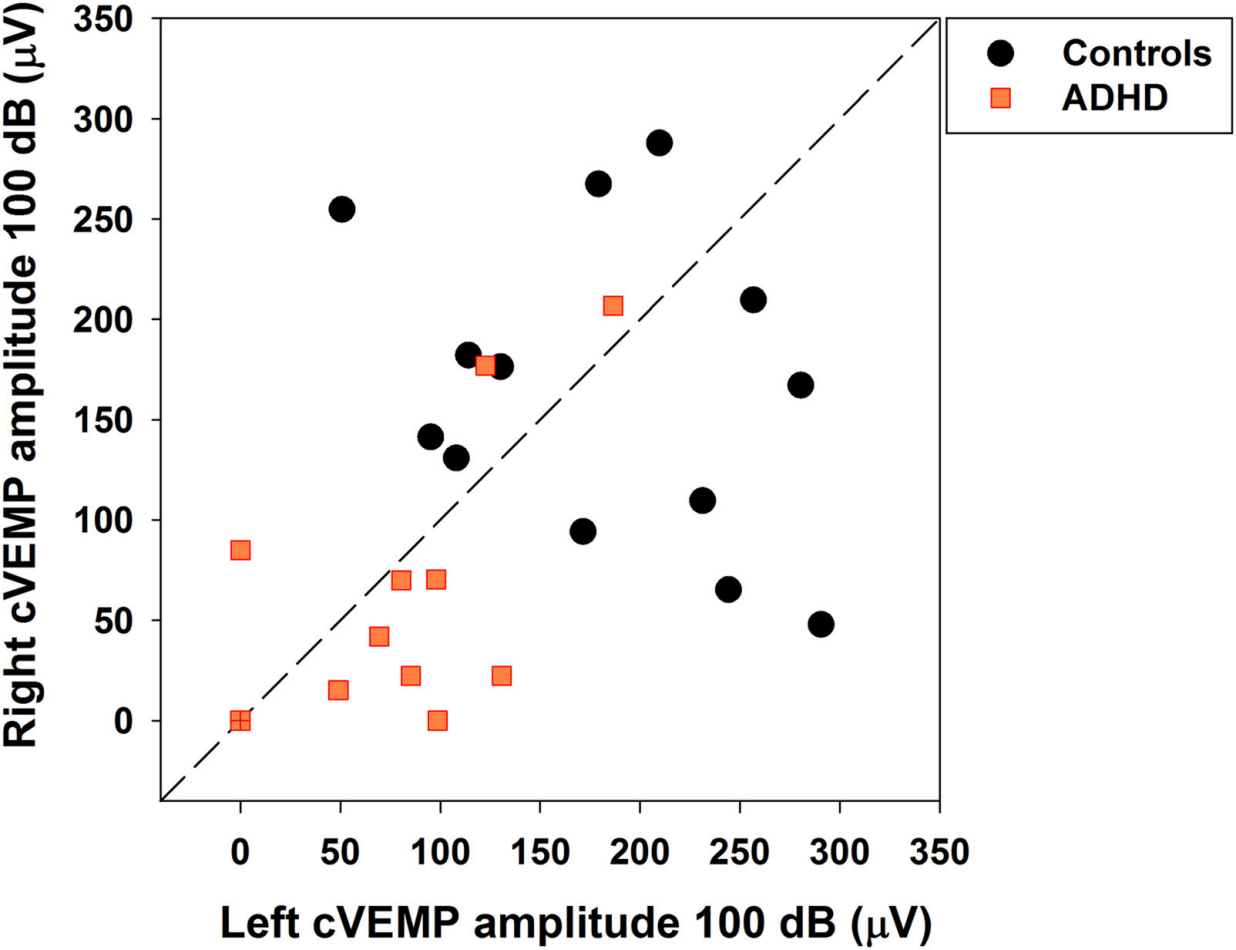
**Individual cervical vestibular-evoked myogenic potential (cVEMP) amplitudes allow separation of a subset of attention deficit and hyperactivity disorder (ADHD) children**. Orange squares and black circles represent individual right and left cVEMP amplitudes in ADHD and control children, respectively. Note that a criterion of left cVEMP amplitude <150 μV and right cVEMP amplitude <100 μV permits identification of ADHD children with 100% specificity and 84.6% sensitivity. The orange symbol at (0,0) with a red cross represents the three ADHD cases with absent cVEMP responses.

Bilateral cVEMPs obtained with 500 Hz tone presented at 100 dB were significantly reduced in ADHD children compared to control children (Figure 4; left cVEMPs: ADHD median cVEMP: 80.4 μV, IQR: 0.0–110.7 μV; control median cVEMP: 179.2 μV, IQR: 111.1–250.6 μV; Mann–Whitney *U* Statistic = 23.0, *p* = 0.002; right cVEMPs: ADHD median cVEMP: 22.4 μV, IQR: 0.0–77.6 μV; control median cVEMP: 167.2 μV, IQR: 101.9–232.3 μV; Mann–Whitney *U* Statistic = 23.0, *p* = 0.002). Remarkably, in 3 of the 13 ADHD children the cVEMP responses were bilaterally absent, while cVEMP responses were always obtained in the control group. On the other hand, there were no significant differences in P1 latencies between ADHD and controls groups (Figure 5). Figure 6 shows the individual analysis of cVEMP amplitude in ADHD and control groups. Using an amplitude criterion of <150 μV in left and <100 μV in right cVEMP responses, we can correctly classify 11 out of 13 ADHD children with 100% specificity.

Next, we studied possible correlations between cVEMP amplitudes and age and height. We found non-significant correlations between age and individual average cVEMP amplitudes (left + right cVEMP amplitudes/2) in the control group (*R* = −0.094, *p* = 0.751) and in the ADHD group (*R* = 0.414, *p* = 0.154). Similarly, we found no significant correlations between individual average cVEMP amplitudes and height in the control (*R* = −0.093, *p* = 0.751) and ADHD (*R* = 0.194, *p* = 0.557) groups.

**Figure 7:**
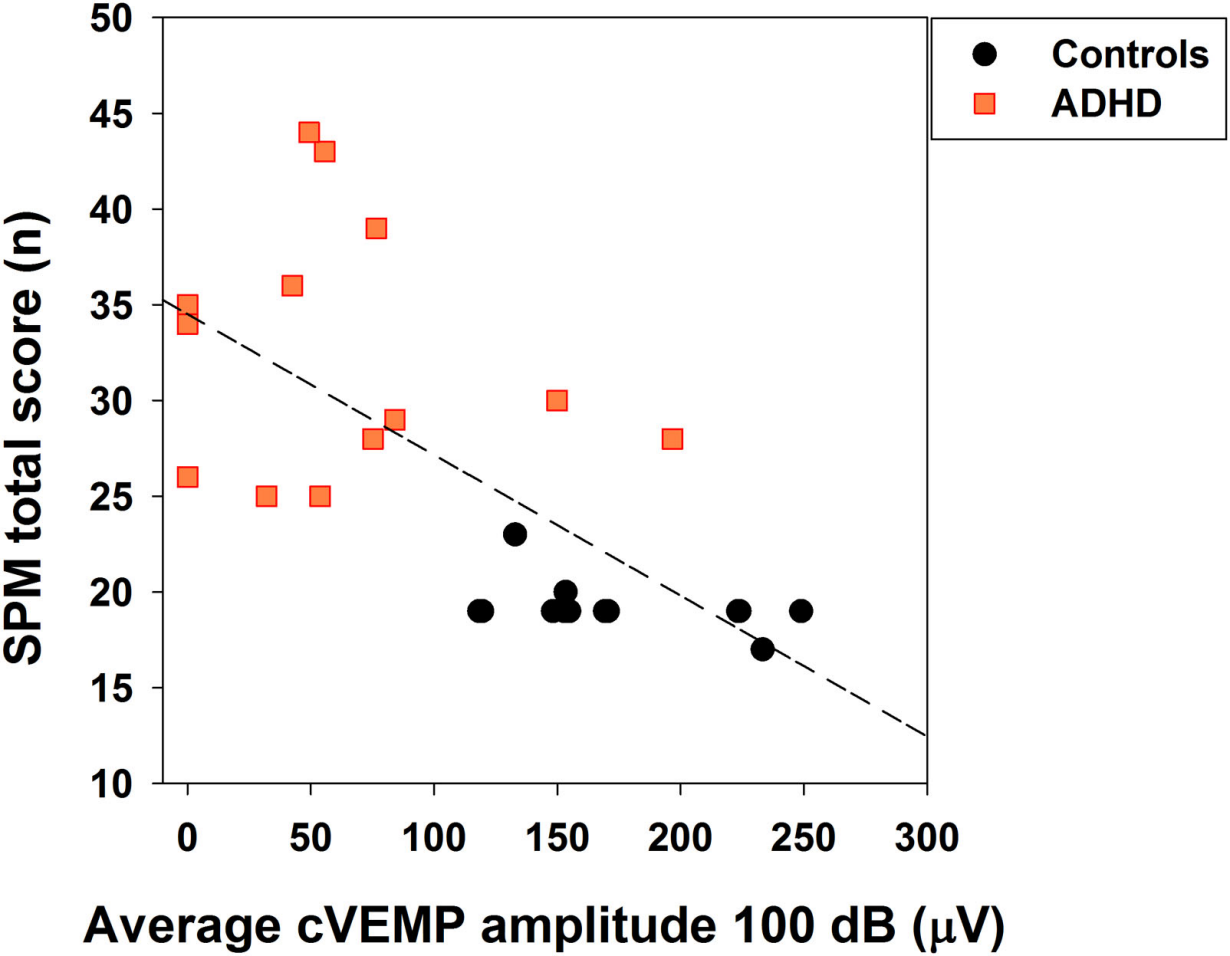
**Significant correlation between the individual average of bilateral cervical vestibular-evoked myogenic potential (cVEMP) amplitudes and behavioral sensory processing measure (SPM) score**. Orange squares and black circles represent the individual average of bilateral cVEMP amplitudes in attention deficit and hyperactivity disorder (ADHD) (*n* = 13) and control children (*n* = 13), respectively. Note that there is an overlap in children controls with 19 points in the SPM score and near 150 μV in cVEMP amplitude. A significant negative correlation was obtained (Spearman, *p* < 0.001), linear regression equation: *f*(*x*) = 34.52 − 0.07 × *x*.

Finally, we studied a possible relation between cVEMP responses and the behavioral symptoms severity using the SPM score. We found a significant negative correlation between the individual average amplitude of bilateral cVEMPs and total SPM score [Spearman, *R*_(26)_ = −0.719, *p* < 0.001; Figure 7], showing that cVEMP amplitudes <100 μV correlate with SPM scores higher than 25 points.

## Discussion

The main findings of this study are (i) no differences in SVV perception between controls and ADHD children, (ii) lower DGI scores and LOS in ADHD patients, and (iii) cVEMP amplitude reduction in a subset of ADHD children. Together, these results highlight the importance of including balance and vestibular assessments in the clinical evaluation of ADHD patients.

### Subjective Visual Vertical

In our study, controls and ADHD children showed no differences in their ability to correctly orient the SVV during the bucket test. These results could be explained through the use of visual and somesthetic cues, for instance, Lee and Aronson (29) found that for infants, visual inputs would be more heavily used than other inputs to control posture and vertical alignment. This might explain why no SVV differences were found between groups. Children learn to rely more on visual information to construct the subjective vertical before integrating vestibular otolith input at a cortical level (19). ADHD children might learn to compensate for deficits in otolith input with visual and somesthetic processing, allowing for a precise visual vertical perception (including those children with absent VEMP reflexes). It is important to highlight the fact that the SVV has been associated more to utricle input than saccule function, which is being assessed through cVEMPs (30). Therefore, the different results obtained with SVV and cVEMPs in ADHD patients could be attributed to a specific dysfunction in saccular pathways in ADHD children.

In healthy adults, the range of absolute deviations of SVV (from vertical 0°) using the bucket test has been reported with values around 0.9 ± 0.7° (mean ± SD) (21). Results from our control group of children and from ADHD group showed mean values of 0.3 ± 0.3° and 0.4 ± 0.1°, correspondingly (mean ± SD). These results suggest that children are as precise in perceiving the SVV or even better than adults. These results contrast with a study on SVV perception in healthy children that showed more variable and less precise SVV values in children with respect to adults (19). However, these authors did not use the bucket test to measure SVV values, instead they placed the children on a platform in a darkened room projecting in front (2 m away) a long figure of a clown representing the vertical line, and children were instructed to use a remote control to straighten the clown up, repeating this task under different visual conditions. These authors relate the variability in their results to a less achieved maturation of the cortical processes involved in the perception of verticality and to limited attention. In our study, as we used the bucket test for the SVV and children would stand in front with the rims of the bucket limiting surrounding visibility, attention to the task was more easily achieved, minimizing variability in their responses. In addition, the examiner rotated the bucket, thus eliminating any motor demands needed from the children.

Further research on the subjective vertical development and perception is needed to identify importance of each sensory system involved at different ages. Varying proprioception and/or postural alignment during the bucket test might offer more insight as to what degree this ability relies on somesthetic input (e.g., using an unstable surface during the test such as a foam platform to stand on).

### Dynamic Gait Index

Significant differences were found in total DGI score, due to particular difficulties in performance for tasks 2, 3, 4, and 6 in ADHD children. For controls, DGI tasks seemed quite simple to perform, with only a few occasional “mild” errors associated to slowing down gait speed while performing tasks. Most controls ranging from ages 8 to 10 obtained the maximum of 24 points, while the range of younger children aged 5–7 tended to slow down gait speed particularly in tasks 3 and 4. These results are in agreement with findings by Lubetzky-Vilnai et al. (26), which assessed DGI in normal children and found mild changes in gait speed during task 3 and 4 were normal in children younger than 12 years. Dissociation of head movements during gait is related to vestibular–proprioceptive function. Due to control results and previous study reports, mild changes in this area are more common at a young age suggesting that maturation process for this function may not yet be complete in ADHD children.

Attention deficit and hyperactivity disorder children not only showed more frequent errors but also most of these would fall under the category of “moderate” changes in gait, indicating more visible disruptions in gait pattern, direction, and speed. In the younger age range, 5–7 years, every ADHD child altered their gait during task 3, and only one managed to perform task 4 without changes in gait pattern. Another observation made during this test is that children with ADHD showed task disruptions not seen in controls, for example, in task 6 tripping over the obstacle, and in task 3 deviating gait directions with head movement. It is important to note that none in the ADHD group achieved the maximum score in the DGI, and that an altered DGI is not a reliable measure of a vestibular dysfunction (31). Therefore, the abnormal gait performance in ADHD children can be consequence of altered motor, cerebellar, proprioceptive, and/or vestibular functions.

### Posturography

The aim of this assessment was to evaluate postural control and static balance abilities with and without visual aid. All tasks were performed under either occluded vision (black screen) or altered visual reality (distractors). This was a challenging test for all children, since it required a large amount of sustained physical (resistance) and mental effort (concentration). Our results show that ADHD children presented more difficulties (larger COP areas and SW velocities) than controls, while keeping balance with both feet on platform with black screen (task 1) and when balancing on one foot (non-dominant side) with black screen (task 3). Similarly, LOS areas were significantly smaller (worse) in the ADHD group, indicating less freedom of body center oscillation before losing balance. This might translate into a lack of postural control and stability during body movements reflected in a higher probability of staggering, falling, and clumsiness in ADHD children. The positive correlations between LOS and age and height in the control group and the lack of correlation in the ADHD group could suggest a delayed or interfered development of posture control in ADHD children.

In tasks 1–8, children were required to maintain their standing posture stable and as still as possible. Controls were able to sway their center of mass with lower speeds and to keep their COP area smaller than the group of ADHD children. These findings are consistent with previous work that used posturography in ADHD children, where surface areas for COP and SWs in ADHD children were significantly larger and higher than those observed in controls (9, 32). In our work, in task 9, COP areas were found to be larger for controls in comparison to ADHD children. A speculative explanation is that in this last task, children were required to explore a virtual surrounding through head and trunk movements while keeping their feet steady. COP areas were significantly larger in controls as they were able to move their center of mass more freely within a wider range reflecting a secure sense of stability. ADHD children showed limited movement in this task, perhaps because of a lack of sense of stability. An important observation made during these static balance tasks was that while controls would often also lose their control of balance, postural reactions were very quick and efficient in these children, allowing them to catch themselves before falling (i.e., moving their feet, shifting body weight, and using their arm movements to regain balance) and were then able to include strategies to avoid further imbalances (i.e., curling one foot around opposite ankle or leg, bending knees slightly, contracting trunk muscles, and opening their arms to the side). On the other hand, ADHD children were much more inefficient in reacting to imbalances, to the point where most of them would even fall off the platform. These observations are supported by the increased number of falls off the platform by ADHD children during posturography reported by Buderath et al. (12).

A speculative explanation is that when directed back to task, they would not elaborate any strategy or new movement that would prevent another fall. In addition, it is important to remark that none of the control children reached the point of falling off the platform. All control children managed to complete the task sequence fluently without interruptions; however, some ADHD children tended to complain they felt tired and some needed a short break half way through the test before continuing. This might reflect that overall for the ADHD group this test was more challenging and required much more effort, because of a lack of postural control abilities, or concentration abilities, or both. Although we cannot rule out the possibility that the poor performance in balance tasks in ADHD children is also influenced by inattention and hyperactivity, these behavioral symptoms should not affect objective measures of otolith function like cVEMP responses.

### Cervical Vestibular-Evoked Myogenic Potential

The cVEMP is an objective measure of a brainstem reflex, which comprise an ipsilateral neural circuit, including the saccule, inferior vestibular nerve, vestibular nuclei, medial vestibulospinal tract, accessory nucleus and nerve, and the SCM muscle (33). The cVEMP waveform corresponds to a compound and averaged myogenic response (known as p13–n23 or P1–N1 response), which can be evoked by high intensity sounds or vibrations (34). The amplitude of cVEMPs is reduced with aging (35, 36) but are unaffected by gender (37). This evoked response has become a clinical standard for otolith (saccular) testing, used in a variety of vestibular disorders (34, 38–41).

Here, we found that an important subset of children diagnosed with ADHD, and without any other neuropsychiatric comorbidity or pharmacological treatment, have altered saccular reflexes measured with cVEMPs. The amplitude reduction of cVEMPs in ADHD children reflects an alteration in the receptor or in the neural circuit that generates cVEMP responses. Moreover, cVEMP amplitudes correlated with high SPM scores, and ADHD children had low DGI values and reduced LOS areas. Therefore, the otolith dysfunction could be contributing to deficits in postural and balance performance in ADHD children (7–9, 11, 12, 32).

The gravisensing otolith organs make direct and indirect connections with the flocculus and the nodulus in the cerebellum, which constitute the vestibulo–cerebellum (42, 43). Regarding the dopamine contribution to cerebellar circuits, there is evidence that those cerebellar areas found to be smaller in ADHD children are parts of the cerebellum that receive large amounts of dopaminergic innervation (44). A speculative proposition is that the smaller cerebellum volumes observed in ADHD children (3, 11) could also be in part due to a deficit of vestibular otolith input. Importantly, a study performed by Bucci et al. (9) reported that postural control abilities in ADHD children improved after 1 month of methylphenidate treatment. An open question is whether cVEMPs responses in this subset of ADHD children can be normalized, if so would this increase in amplitude be through methylphenidate treatment or vestibular rehabilitation therapies?

The vestibular inputs contribute to a variety of cognitive processes including visuospatial ability, memory, attention, and executive functions (45). Projections from the vestibular organs toward the hippocampus and cortical centers involved in memory and spatial orientation may represent the neural basis for the association between vestibular and cognitive function. In this line, a recent study found altered cVEMP responses in aged adults with cognitive impairment or dementia (46). We propose that altered cVEMP responses reflect a vestibular impairment that contributes to the ADHD phenotype, and consequently, the vestibular pathways should be added to the brain network affected in ADHD patients (5).

Since cVEMPs of reduced amplitudes correlated with dysfunctional behaviors assessed with the SPM form, some open questions are whether the presence of ADHD-like symptoms indicates possible vestibular dysfunction? Are altered saccular reflexes a part of the neural basis of ADHD disorders? Another possibility is that vestibular alterations constitute a completely different diagnosis, which tends to present itself with similar ADHD behaviors in the pediatric population? In the present work, we are not able to answer these questions, but we show that balance and otolith assessments are important to consider in the clinical evaluation of ADHD patients.

## Conclusion

In this study, the most novel result is that cVEMP amplitudes are reduced in ADHD children. Moreover, 11 out of 13 ADHD children could be classified with 100% specificity using a cVEMP amplitude criterion. These findings suggest that vestibular otolith function is altered in a significant number of children exhibiting ADHD symptoms. Future studies in utricular and semicircular canal functions using the video head impulse test and the possible pharmacological modulation of cVEMP responses are needed for a more complete understanding of a vestibular dysfunction in ADHD children.

## Author Contribution

VI and PD designed research; VI and DO performed research; VI, DO, FA, and PD analyzed data; VI, FA, and PD wrote the manuscript.

## Conflict of Interest Statement

The authors declare that the research was conducted in the absence of any commercial or financial relationships that could be construed as a potential conflict of interest.
